# Alcohol use‐specific treatment initiation among patients undergoing surgical procedures: A retrospective cohort analysis

**DOI:** 10.1111/acer.70231

**Published:** 2026-01-12

**Authors:** Megan L. Rolfzen, Matt Muellner, Sarah V. Mattioli, A. Jerrod Anzalone, Anne C. Fernandez, Anne P. Ehlers, Karsten Bartels

**Affiliations:** ^1^ Department of Anesthesiology University of Michigan Ann Arbor Michigan USA; ^2^ Department of Anesthesiology University of Nebraska Medical Center Omaha Nebraska USA; ^3^ SporeData, Inc. Durham North Carolina USA; ^4^ Department of Biostatistics, College of Public Health University of Nebraska Medical Center Omaha Nebraska USA; ^5^ Department of Psychiatry University of Michigan Ann Arbor Michigan USA; ^6^ Center for Healthcare Outcomes & Policy University of Michigan Ann Arbor Michigan USA; ^7^ Department of Surgery University of Michigan Ann Arbor Michigan USA

**Keywords:** alcohol, alcohol screening, AUDIT‐C, health services, perioperative, surgery

## Abstract

**Background:**

Alcohol use is common in surgical patients and linked to morbidity and mortality. Yet, alcohol screening and treatment are frequently overlooked in perioperative care. This study examines how patient risk for unhealthy alcohol use is associated with the likelihood of receiving any treatment for the purpose of alcohol risk reduction or cessation.

**Methods:**

All records from surgical patients with quantifiable Alcohol Use Disorders Identification Test—Consumption (AUDIT‐C) scores in the *All of Us* Research Program, a precision medicine‐focused database harmonizing surveys and electronic health records, were evaluated. The association between treatment for at‐risk alcohol use, including psychotherapy and pharmacologic therapy, and categorical AUDIT‐C risk was estimated using adjusted multivariable logistic regression models.

**Results:**

Any alcohol use treatments were initiated in 0.5% of patients within 90 days of a procedure. Patients in high‐risk and severe‐risk AUDIT‐C groups had significantly increased odds of receiving any treatment (aOR 2.37, 95% CI 1.06, 4.74; aOR 10.1, 95% CI 6.02, 16.8). Similarly, participants with an alcohol use disorder (AUD) diagnosis were nine times more likely to receive any treatment than those without a diagnosis (aOR 9.33, 95% CI 5.97, 14.70). Yet only 0.7% of high‐risk, 4% of severe‐risk AUDIT‐C participants, and 1.7% of participants diagnosed with AUD received any treatment.

**Conclusions:**

Surgical patients with identified severe risk for unhealthy alcohol consumption are more likely to receive perioperative alcohol‐specific treatment. However, even for high‐risk patients, the provision of perioperative treatments to reduce alcohol intake is rare. Future work should focus on overcoming barriers to reduce unhealthy alcohol use after surgery.

## Graphical abstract

Although surgical patients with higher Alcohol Use Disorders Identification Test (AUDIT‐C) risk scores have increased odds of receiving treatment to reduce alcohol use, overall treatment rates within 90 days before or after surgery are exceedingly low—less than 5% in the severe‐risk group (AUDIT‐C score 8–12). A significant gap exists between identified alcohol use risks and treatment in perioperative care. More widespread, effective implementation of targeted screening and intervention strategies is needed to address unhealthy alcohol use among surgical patients.

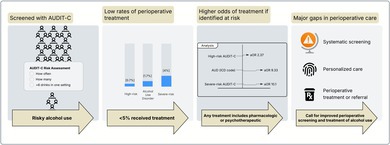

## INTRODUCTION

Unhealthy alcohol use poses a significant public health risk. Unhealthy alcohol use ranges from risky drinking to diagnosed alcohol use disorder (AUD) (Krist & Bradley, 2025). In the United States, alcohol consumption is consistently ranked in the top 10 leading causes of death and disability‐adjusted life years (US Burden of Disease Collaborators et al., 2018). As a preventable and modifiable risk factor for surgical morbidity, cancer, liver disease, and other detrimental health outcomes, alcohol use represents a high‐priority area for public health action (National Institute on Alcohol Abuse and Alcoholism (NIAAA), 2021a; Rolfzen et al., 2022; Spadola et al., 2015). However, the AUD epidemic is outpacing the supply of specialized health professionals, especially since the COVID‐19 pandemic, when social disruption incited an increase in substance use, including alcohol (Ayyala‐Somayajula et al., 2024; Bantounou, 2023). For patients unlikely to pursue routine primary care, a surgical encounter may be one of few opportunities to engage with a healthcare professional. Hospitalizations in general, and surgical episodes in particular, have been found to motivate behavior change related to substance use (Shi & Warner, 2010).

Typically, a full Alcohol Use Disorders Identification Test screening occurs in a primary care setting, but given time and staffing constraints, a shortened 3‐question version of the validated risk assessment, the Alcohol Use Disorders Identification Test—Consumption (AUDIT‐C), can reliably be performed before surgery (Moehring et al., 2019). Perioperative clinicians are uniquely positioned to address the existing screening, identification, and therapy gap for unhealthy alcohol use around the time of surgery (Jenkins et al., 2025; Lane et al., 2024). Incorporating perioperative screening and treatment could reach many Americans, given that one in nine undergoes a procedure yearly (Bicket et al., 2024).

Despite surgical and anesthesia groups' recommendations to routinely screen adult patients for alcohol use (ASA, 2020), screening and treatment remain overlooked in the design of perioperative care plans (Scharwachter et al., 2016). A wide variety of accessible treatments could be offered, including brief intervention, referral to outpatient therapy, or one of three FDA‐approved medications for alcohol use disorder: naltrexone, disulfiram, and acamprosate (McPheeters et al., 2023; NIAAA, 2021b). By leveraging improved screening practices to identify patients who do not meet alcohol use disorder criteria but engage in high‐risk alcohol consumption patterns, perioperative clinicians may be able to alter the postoperative trajectory (Bartels & Schacht, 2021; Budworth et al., 2019).

The extent to which alcohol use screening and treatment occur perioperatively, especially since the COVID‐19 pandemic resulted in an increasingly strained healthcare system, is unknown. Our objective in this observational study of a large, US‐based multicenter longitudinal cohort, was to quantitatively analyze how the risk for unhealthy alcohol use influences the provision of alcohol‐specific treatments in the perioperative setting. The data source, *All of Us* Research Program, offers unique advantages in that it links standardized AUDIT‐C screening data with real‐world electronic health record outcomes in a highly representative sample. Our primary aims were to:

1. describe contemporary treatment patterns in the 90 days periprocedurally in patients screened for unhealthy alcohol use. We hypothesized that the rates for receiving any treatment (psychotherapy or pharmacotherapy) specific to reducing alcohol use around the time of surgery would be low (likely less than 5%);
2. identify factors associated with any alcohol use‐related treatment offerings. We hypothesized that the provision of alcohol‐specific treatment to patients undergoing a procedure would be dependent on distinct patient and clinician factors.

## METHODS

### Study design

This study utilized the *All of Us* Research Program Controlled Tier, version 8, which included a limited dataset. The National Institutes of Health (NIH) Institutional Review Board has determined that research using this dataset represents nonhuman subject research. The University of Michigan and the University of Nebraska Medical Center both hold an institutional Data Use and Registration Agreement with the NIH *All of Us* Research Program. This study is reported as a retrospective cohort study, adhering to the Strengthening the Reporting of Observational Studies in Epidemiology (STROBE) guidelines and the Checklist for Statistical Assessment of Medical Papers (CHAMP) statement guidelines (Appendix S1) (Mansournia et al., 2021; von Elm et al., 2008). Statistical plans were registered on Open Science Framework before data analysis (https://doi.org/10.17605/OSF.IO/WDY5K).

### Data source

Records from May 2018 to October 2023 contained in the *All of Us* Research Program from inpatients and outpatients undergoing a surgical procedure aged 18 years or older, who received standardized screening for unhealthy alcohol use using the AUDIT‐C within 3 years of surgery, were included. The *All of Us* Research Program is a nationwide initiative led by the NIH that integrates clinical, survey, and genomic data from consented participants as a critical component of the Precision Medicine Initiative (Denny et al., 2019). The database contains standardized research (Case Report Forms) and real‐world (imported from Electronic Health Records) data for a diverse range of participants, making it a unique, inclusive, cloud‐based platform that was designed to make research Findable, Accessible, Interoperable, and Reusable (FAIR) (Mayer & Huser, 2023; Ramirez et al., 2022). Compared to other surgical registries or institutional databases, preliminary characterization of the surgical cohort within the *All of Us* Research Program reveals a larger, more racially diverse, and potentially lower‐risk surgical population (Douville et al., 2025).

### Exposures

The primary exposure variable was the AUDIT‐C screen, a validated measure to identify people with unhealthy drinking patterns who might benefit from brief care interventions (Rubinsky et al., 2012). We used responses to the validated AUDIT‐C screening questions, derived from the lifestyle survey mapped to Observational Medical Outcomes Partnership (OMOP), to classify patients into risk categories stratified by gender (Appendix S1: Table A.1). For men, AUDIT‐C scores were categorized as: low risk (0–3), moderate risk (4–5), high risk (6–7), and severe risk (8–12). For women, the categories were: low risk (0–2), moderate risk (3–5), high risk (6–7), and severe risk (8–12). Gender‐specific item modifications were made based on prior proposals to address binge drinking (Bradley et al., 2007; Neumann et al., 2012; Office of Quality and Patient Safety, 2021). If the gender was unknown, missing, or self‐reported as “other,” the AUDIT‐C score cutoffs were conservatively categorized based on the male gender grouping. A score of 8 or higher has been associated with mortality and trauma‐related hospitalization (Williams et al., 2012); however, any score suggesting a moderate risk alcohol use pattern or greater is indicative of unhealthy alcohol use (Bradley et al., 2007). Here, we incorporate any screen with a score greater than 0 to fully evaluate the extent to which current screening measures inform treatment paradigms. AUDIT‐C surveys were considered temporally relevant if they were completed within 3 years prior to the recorded procedure date. A secondary independent variable of interest was a formal alcohol use disorder diagnosis, identified by International Classification of Diseases, Tenth Revision (ICD‐10) coding in the electronic health record (Appendix S1: Table A.2).

### Outcomes and covariates

The primary outcome was the provision of any treatment for at‐risk alcohol use offered within 90 days before and after the procedure date. For brevity, we use the term “alcohol use treatment” as a shorthand to represent any pharmacologic or behavioral therapies that were utilized for the purpose of alcohol risk reduction or cessation. Alcohol‐specific treatments were categorized into (1) psychotherapy: cognitive and behavioral therapy, rehabilitation, or referral to outpatient behavioral treatment, and (2) pharmacotherapy: in‐hospital and/or 90‐day postprocedure naltrexone, acamprosate, or disulfiram, which are Food and Drug Administration (FDA)‐approved pharmaceutical treatments for alcohol use disorder. Procedures were defined according to Current Procedural Terminology (CPT) codes, adapted to the OMOP Common Data Model, and categorized based on keywords in the procedure name, similar to previously published work (Aminpour et al., 2025; Douville et al., 2025). Alcohol treatments provided within 90 days of the procedure date were collected by mapping RxNorm and ICD‐10‐CM codes to specific OMOP treatment identifiers (tables available upon request). In patients with multiple surgeries, the first procedure available was used for analysis. To retain the low incidence counts in our primary outcome of interest, we received an exemption from the Data and Statistics Dissemination Policy by the *All of Us* Resource Access Board.

Sociodemographic variables were obtained from the *All of Us* tables containing Participant Provided Information (PPI) and Logical Observation Identifiers Names and Codes (LOINC) terminologies, which integrate area‐level measures from the Social Determinants of Health and American Community surveys (Appendix S1: Table A.3). Variables that reflect the socioeconomic characteristics of participants' residential areas include: (1) the percentage of individuals living below the federal poverty level; (2) the median household income in the past 12 months, adjusted to 2015 US dollars; (3) the percentage of households receiving public assistance or Supplemental Nutrition Assistance Program benefits in the prior 12 months; (4) the fraction of the population without health insurance coverage; (5) the fraction of vacant housing units; and (6) the fraction of adults aged 25 and older with at least a high school education (including General Education Development equivalency). Additionally, a nationwide Community Deprivation Index was included as a summary measure of “community deprivation,” ranging from 0 to 1, with higher values indicating greater deprivation (Acharya & Natarajan, 2024; Brokamp et al., 2019).

Patient and surgical encounter‐level characteristics were included as covariates for descriptive purposes and confounder adjustment of variables potentially associated with alcohol use and treatment. We selected potential confounders using a combination of clinical judgment and evidence from the literature (Lee, 2014). In addition to demographic patient‐level characteristics such as age, gender (self‐reported survey response), race, and ethnicity, we adjusted for several socioeconomic and clinical factors, including insurance status, education level, comorbidities (captured through the Charlson Comorbidity Index and its components) (Quan et al., 2011) mental health conditions (e.g., anxiety and depressive disorders), surgical specialty, length of stay, and area‐level indicators such as poverty level, median household income, assisted income, uninsured population, and housing vacancy (Appendix S1: Table A.3). These variables were included in the models to account for potential confounding in the association between AUDIT‐C risk level and receipt of alcohol treatment.

### Statistical analyses

The data were visualized with frequency distributions, percentages, and near‐zero variance for categorical variables (e.g., gender, race, ethnicity, insurance, visit classification, anxiety, depressive disorder, rural residence, and type of surgery), as well as the distribution of numeric variables (e.g., age, length of stay, total AUDIT‐C score, Charlson Comorbidity Index, and number of alcohol treatments provided). Near‐zero variance, which refers to categorical variables with very low variability across the sample, was addressed by recategorizing or collapsing levels as needed. Descriptive statistics summarizing demographic and clinical characteristics were described with mean ± standard deviation (SD), median [interquartile range; IQR], and numbers/proportions, as appropriate. We further explored variable relationships using correlation matrices and visual plots. For numeric, ordinal, and logical items, we applied Pearson, polychoric, or polyserial correlations, as appropriate. We evaluated multicollinearity by examining correlation coefficients, which reflect the degree of association between pairs of variables through matrices. This analysis allowed us to identify and remove redundant variables with high correlations exceeding 0.9 (Appendix S1: Figure A.1). We analyzed patterns of missing data and observed low overall missingness (Table 1). We did not perform a complete case analysis due to the nonrandom missingness in surveys related to background characteristics (Cronin et al., 2023).

**TABLE 1:** Demographic and clinical characteristics of procedural patients in the *All of Us* Research Program stratified by AUDIT‐C risk assessment.

Variable	Total, *N* (%)	Low risk^a^, *N* (%)	Moderate risk, *N* (%)	High risk, *N* (%)	Severe risk, *N* (%)	Missing (%)
Overall	21,352 (100)	14,210 (66.6)	5,015 (23.5)	1,257 (5.9)	870 (4.1)	
Age (mean ± SD)	53.1 (16.6)	54.3 (16.3)	51.7 (17.6)	47.9 (15.5)	48.5 (13.7)	0
Gender						
Male	8,424 (39.5)	5,545 (39.0)	1,628 (32.5)	66^*^	58^*^	0
Female	12,527 (58.7)	8,370 (58.9)	3,321 (66.2)	566 (45.0)	270 (31.0)	
Other	401 (1.9)	295 (2.1)	66 (1.3)	2^*^	<20	
Race						
White	10,794 (50.6)	7,092 (49.9)	2,808 (56.0)	551 (43.8)	343 (39.4)	0
Black	4,802 (22.5)	3,232 (22.7)	944 (18.8)	345 (27.4)	281 (32.3)	
Other	5,756 (27.0)	3,886 (27.3)	1,263 (25.2)	361 (28.7)	246 (28.3)	
Ethnicity						
Not Hispanic	16,714 (78.3)	11,113 (78.2)	3,967 (79.1)	952 (75.7)	682 (78.4)	0
Hispanic	4,007 (18.8)	2,659 (18.7)	914 (18.2)	269 (21.4)	165 (19.0)	
Other	631 (3.0)	438 (3.1)	134 (2.7)	36 (2.9)	23 (2.6)	
Education^b^						
College+	14,175 (66.4)	9,521 (67.0)	3,597 (71.7)	683 (54.3)	374 (43.0)	0
No college	7177 (33.6)	4689 (33.0)	1,418 (28.3)	574 (45.7)	496 (57.0)	
Insurance						
Medicaid	3,235 (15.2)	2,131 (15.0)	640 (12.8)	256 (20.4)	208 (23.9)	0.1
Medicare	2945 (13.8)	2,138 (15.1)	630 (12.6)	97 (7.8)	80 (9.2)	
Other	15,070 (71.0)	9,941 (69.9)	3,745 (74.6)	904 (71.8)	582 (66.9)	
Socioeconomic (median [IQR])						
Poverty level^c^	16.48 [12.72, 20.59]	15.74 [12.53, 20.41]	16.48 [12.38, 20.59]	17.75 [14.25, 20.59]	17.75 [15.74, 20.59]	4.3
Median household income ($)^d^	61,192.64 [55,344.42, 74,163.24]	61,192.64 [55,344.42, 74,084.32]	61,580.82 [56,305.95, 78,108.85]	61,024.19 [55,344.42, 71,783.36]	61,580.82 [55,344.42, 71,783.36]	4.3
Assisted income^e^	15.82 [11.58, 19.96]	15.38 [11.58, 19.18]	15.27 [11.36, 22.05]	16.19 [13.02, 22.05]	16.19 [14.55, 18.51]	4.3
Uninsured fraction^f^	10.02 [6.35, 12.87]	10.02 [6.19, 12.87]	9.54 [6.38, 12.87]	10.30 [6.86, 12.87]	10.30 [5.74, 12.87]	4.3
Vacant housing^g^	10.84 [6.24, 12.36]	10.84 [6.57, 12.36]	10.44 [6.20, 12.20]	11.39 [6.62, 12.39]	11.39 [6.62, 13.87]	4.3
Deprivation Index	0.33 [0.29, 0.39]	0.33 [0.29, 0.39]	0.32 [0.28, 0.39]	0.33 [0.30, 0.39]	0.33 [0.30, 0.39]	4.3
Medical comorbidity						
Anxiety	5,643 (26.4)	4,000 (28.1)	1,107 (22.1)	290 (23.1)	246 (28.3)	0
Depression	5,277 (24.7)	3,729 (26.2)	1,003 (20.0)	292 (23.2)	253 (29.1)	
Alcohol use disorder	2,387 (11.2)	1,205 (8.5)	624 (12.4)	255 (20.3)	303 (34.8)	
Charlson Comorbidity Index (mean ± SD)	7.1 (6.1)	7.7 (6.2)	5.7 (5.5)	5.5 (5.40)	5.9 (5.5)	
Surgical specialty						
General surgery	12,440 (58.3)	8,321 (58.6)	2,939 (58.6)	680 (54.1)	500 (57.5)	0
Cardiothoracic surgery	886 (4.1)	620 (4.4)	175 (3.5)	55 (4.4)	36 (4.1)	
Interventional	1,046 (4.9)	727 (5.1)	208 (4.1)	67 (5.3)	44 (5.1)	
Neurosurgery	756 (3.5)	526 (3.7)	172 (3.4)	<40	<30	
OB/GYN	980 (4.6)	571 (4.0)	306 (6.1)	76 (6.0)	27 (3.1)	
Orthopedic surgery	1,703 (8.0)	1,115 (7.8)	424 (8.5)	97 (7.7)	67 (7.7)	
ENT, OMFS, Ophthalmology	979 (4.6)	642 (4.5)	216 (4.3)	75 (6.0)	46 (5.3)	
Plastic surgery	1385 (6.5)	814 (5.7)	373 (7.4)	114 (9.1)	84 (9.7)	
Vascular surgery	992 (4.6)	742 (5.2)	158 (3.2)	54 (4.3)	38 (4.4)	
Other	185 (0.9)	132 (0.9)	44 (0.9)	<10	<10	
Length of stay (mean ± SD)	3.6 (9.8)	3.8 (10.1)	2.7 (6.6)	3.3 (12.5)	3.9 (14.6)	0

Abbreviations: AUDIT‐C, Alcohol Use Disorders Identification Test—Consumption; ENT, ear, nose, throat; IQR, interquartile range; OB/GYN, obstetrics and gynecology; OMFS, oral and maxillofacial surgery; SD, standard deviation.

^*^ Ambiguity related to a category with value <20.

^a^ Risk categories are described using cutoffs based on gender: low risk (F: 0–2; M: 0–3), moderate risk (F: 3–5; M: 4–5), high risk (6–7), severe risk (8–12).

^b^ College+ refers to completing a college degree or more.

^c^ Fraction of population with income in past 12 months below poverty level.

^d^ Median household income in the past 12 months in 2015 inflation‐adjusted dollars.

^e^ Assisted income is the fraction of households receiving public assistance, food stamps, or Supplemental Nutrition Assistance Program programs.

^f^ Fraction of population with no health insurance coverage.

^g^ Fraction of neighborhood‐level houses that are vacant in the area.

Our primary analysis employed logistic regression to evaluate the association between categorical AUDIT‐C risk level (stratified by gender) and the receipt of any alcohol treatment (psychotherapy and/or pharmacologic therapy). Covariates included for adjustment were demographic variables (age, gender, race, and ethnicity), socioeconomic indicators (education, insurance status, income, area‐level deprivation metrics), comorbidities (Charlson Comorbidity Index, anxiety, and depression), and hospitalization‐level metrics (length of stay and surgical specialty).

We performed all analyses using the R statistical software (©The R Foundation, Bell Laboratories). We present *p*‐values from *t*‐tests (for numeric variables) and chi‐squared tests (for categorical variables), with significance set at *p* < 0.05. We report the association between AUDIT‐C risk level and the outcome as odds ratios (OR) with 95% confidence intervals (CI). Results were considered statistically significant if the CI did not include 1.0.

### Power and sample size

The final sample size was determined by the number of eligible patients in the *All of Us* Research Program dataset. We included patients who underwent a procedure and completed the AUDIT‐C scale within 3 years before the procedure date. The pwrss R package was used to evaluate a logistic regression of the binary dependent variable (any alcohol treatment) and 10 independent variables with a sample size of 10,213 to achieve 80% power at a 0.05 two‐tailed significance level to detect an adjusted odds ratio of 1.2 or greater as significant. Based on prior literature, we assumed that 5% of patients at risk for or diagnosed with AUD receive any treatment, and no significant correlation among predictors (Substance Abuse and Mental Health Services Administration, 2023). Details regarding a post hoc power analysis can be found in Appendix S1.

### Sensitivity analyses and subgroup analyses

To assess the impact of varying inclusion criteria and degrees of alcohol use based on screening tools or formal diagnoses, we performed several sensitivity and subgroup analyses. First, we repeated the primary analysis, excluding cases with a lower than moderate risk for risky alcohol use based on the AUDIT‐C screening, and used the moderate risk category as the referent group for both the unadjusted and adjusted models. Additionally, we repeated the primary analysis for three separate subgroup analyses: (1) restricting the alcohol use cohort to patients who were formally diagnosed with an alcohol use disorder via ICD‐10 code, (2) excluding those without an AUD code and low‐risk AUDIT‐C scores, and (3) restricting the cohort to patients screened within the year before the procedure was performed to ascertain patient impact. Accordingly, we report the average time between the AUDIT‐C screen and procedure for the dataset.

## RESULTS

### Descriptive statistics

**FIGURE 1:**
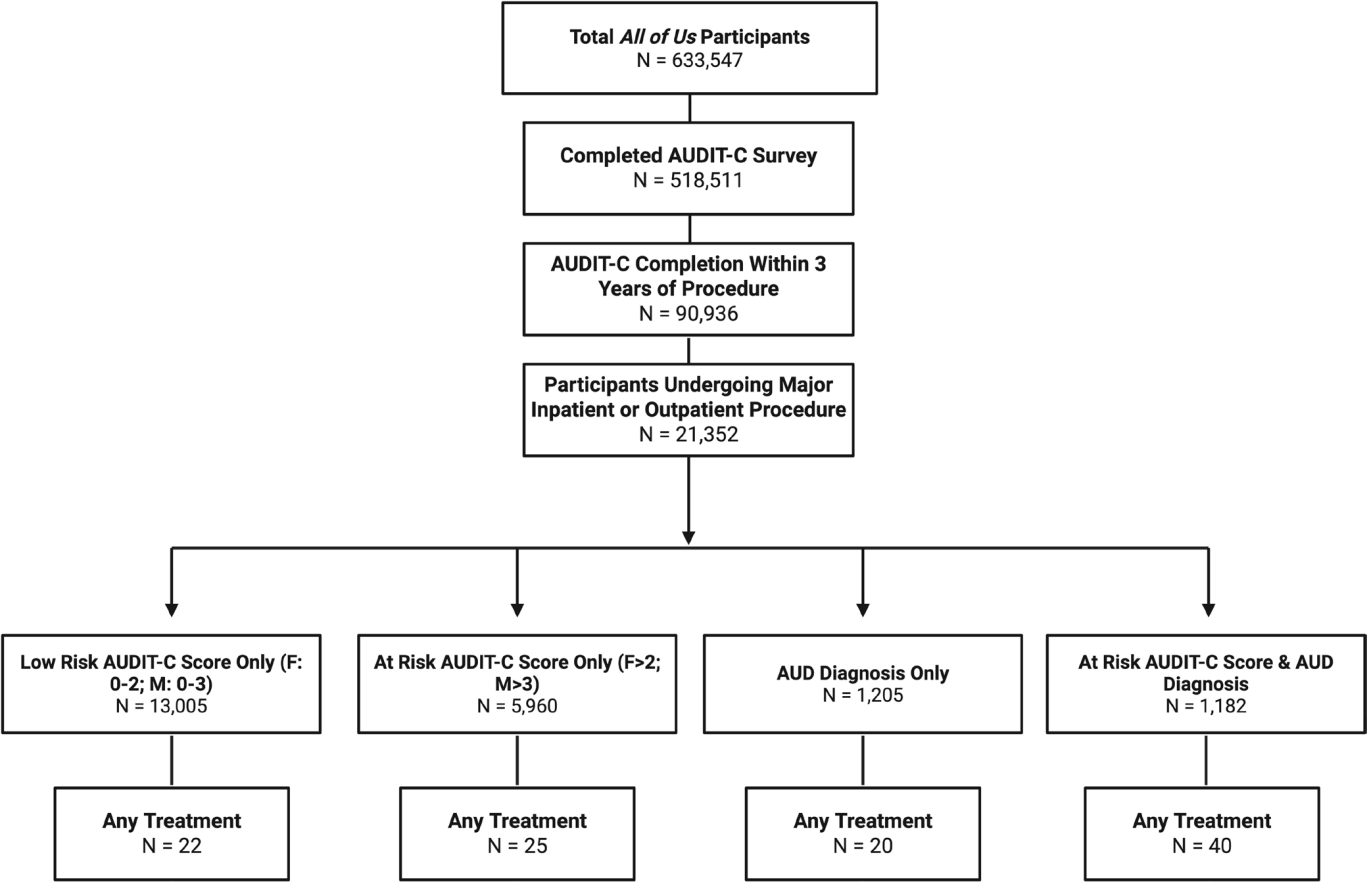
Flow diagram. Patient distribution and treatment types based on AUDIT‐C scores and AUD diagnoses in the *All of Us* dataset. Each category is mutually exclusive.

In total, 633,547 participants in the *All of Us* Research Program database were assessed for eligibility. After filtering for patients who underwent a procedure with a recorded AUDIT‐C score within the previous 3 years, 21,352 patients were included (Figure 1). Of the 21,352 participants who underwent a procedure, 17,297 (81%) were inpatients and 4,055 (19%) were outpatients. Baseline demographic characteristics and univariate analysis of the full cohort are reported in Table 1. The majority of the cohort identified as female (12,527; 58.7%), white race (10,794; 50.6%), attended college or higher (14,175; 66.4%), were not enrolled in Medicaid or Medicare (15,070; 71.0%), screened as low risk on the AUDIT‐C (14,210; 66.6%), and had a mean age of 53 years (standard deviation 16.6). Lower age, male gender, less education, and higher poverty and assisted income levels were all associated with higher risk AUDIT‐C scores. Similarly, several comorbid conditions, including depression and a formal AUD diagnosis, were associated with a higher risk AUDIT‐C category. There were no apparent statistically significant differences in AUDIT‐C by ethnicity or uninsured fraction (Table 1).

According to their AUDIT‐C scores, 23.5%, 5.9%, and 4.1% of surgical patients qualified as moderate, high, and severe risk for unhealthy alcohol use, respectively. Only 0.4%, 0.7%, and 4% of these at‐risk patients received any alcohol treatment. Treatment within 90 days of the procedure was more likely to be initiated in participants in the severe risk group. Overall, pharmacotherapy was utilized more than psychotherapy (Table 2).

**TABLE 2:** Frequency of treatment modalities stratified by AUDIT‐C screening risk categoriess.

Variable	Total, *N* (%)	Low risk, *N* (%)	Moderate risk, *N* (%)	High risk, *N* (%)	Severe risk, *N* (%)
Total, *N* (%)	21,352 (100)	14,210 (66.6)	5,015 (23.5)	1,257 (5.9)	870 (4.1)
Pharmacotherapy	90 (0.4)	33 (0.2)	17 (0.3)	9 (0.7)	31 (3.6)
Psychotherapy	18 (0.1)	9 (0.1)	4 (0.1)	0 (0.0)	5 (0.6)
Any Treatment^a^	107 (0.5)	42 (0.3)	21 (0.4)	9 (0.7)	35 (4.0)

*Note*: Risk categories are described using cutoffs based on gender: low risk (F: 0–2; M: 0–3), moderate risk (F: 3–5; M: 4–5), high risk (6–7), severe risk (8–12).

Abbreviation: AUDIT‐C, Alcohol Use Disorders Identification Test—Consumption.

^a^ Any treatment is a combination of both pharmacotherapy and psychotherapy treatment modalities.

### Multivariable analysis

**FIGURE 2:**
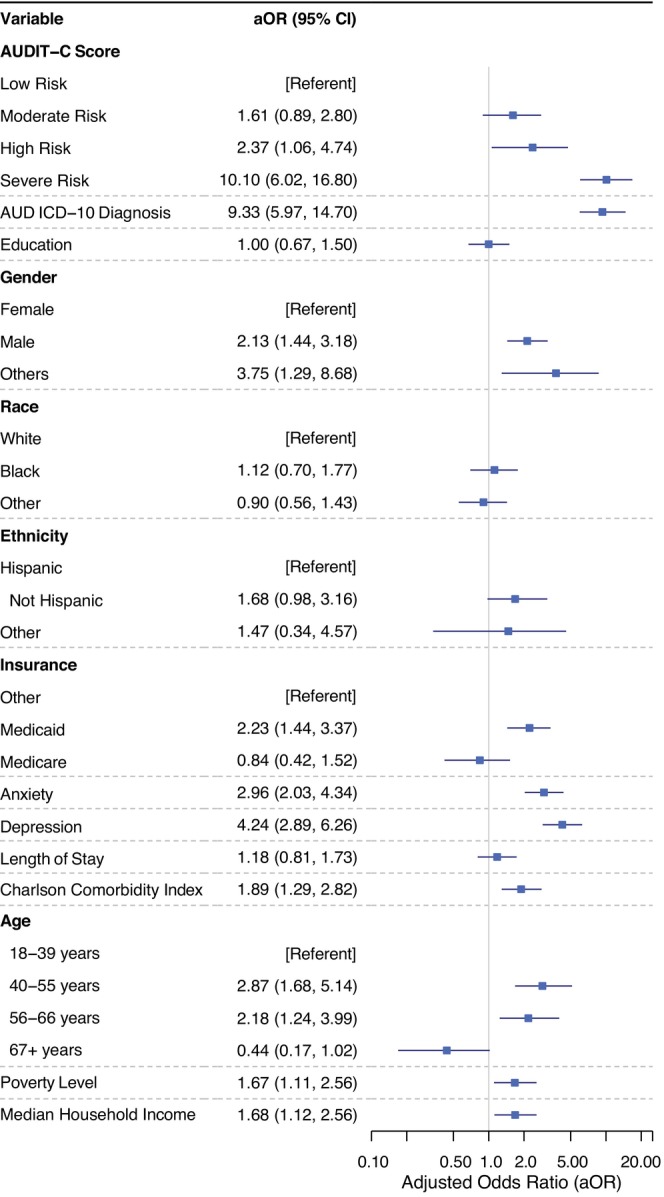
Adjusted odds of receiving any alcohol treatment postprocedurally. Model adjusted for gender, race, ethnicity, insurance status, anxiety, depression, length of stay, surgical specialty, Charlson Comorbidity Index, and neighborhood‐level socioeconomic indicators (age distribution, assisted income, uninsured rate, education level, housing vacancy, poverty rate, and median household income).

Results from multivariable logistic regressions for the primary outcome after adjusting for conditions of interest are summarized in Figure 2. Several factors were independently associated with receiving treatment. Qualifying for high‐risk and severe‐risk AUDIT‐C groups was associated with significantly increased odds of receiving any alcohol treatment (aOR 2.37, 95% CI 1.06, 4.74; aOR 10.1, 95% CI 6.02, 16.80). Participants diagnosed with depression (aOR 4.24, 95% CI 2.89, 6.26) or anxiety (aOR 2.96, 95% CI 2.03, 4.34) were at increased odds of receiving treatment. Male gender (aOR 2.13, 95% CI 1.44, 3.18) and individuals identifying as “other” gender (aOR 3.75, 95% CI 1.29, 8.68) also had increased odds compared to the female gender. Middle‐aged adults (ages 40–66) and those with higher comorbidity burden were at increased odds of receiving treatment. Seemingly, socioeconomic context influenced care delivery. Living in areas with higher median income, lower poverty rates, fewer vacant houses, and higher insurance coverage was associated with increased odds of receiving alcohol treatment. Insurance type was also relevant, with Medicaid coverage associated with higher odds of receiving any treatment (aOR 2.23, 95% CI 1.44–3.37, *p* < 0.001), while Medicare showed no significant association (aOR 0.84, 95% CI 0.42–1.52, *p* = 0.59). Race, ethnicity, length of stay, and high school education were not statistically significant predictors in this model. Compared to general surgery, those undergoing plastic surgery were almost 2.4 times more likely to receive any treatment (aOR 2.37, 95% CI 1.32, 4.02), while other specialties were not associated with alcohol treatment. The unadjusted odds of receiving any alcohol treatment within 90 days of a procedure are reported in Appendix S1: Figure A.2.

### Sensitivity and subgroup analyses

In sensitivity analyses with the moderate risk group as the referent group, the severe risk AUDIT‐C group remained more likely to receive any treatment (aOR 7.00, 95% CI 3.67, 13.70); however, the high‐risk group no longer remained more likely to receive any treatment (aOR 1.63, 95% CI 0.68, 3.64). Across all AUDIT‐C screening categories, participants with an AUD diagnosis were nine times more likely to receive any treatment than those without a diagnosis (aOR 9.33, 95% CI 5.97, 14.70). Participants with an AUD diagnosis and at least moderate risk subgroup based on the AUDIT‐C score were eight times more likely to receive any treatment (aOR 8.39, 95% CI 4.58, 15.70). Subgroup analysis of patients with an AUDIT‐C screen within a year of their procedure yielded similar findings to the primary analysis, with high‐risk and severe‐risk AUDIT‐C groups associated with significantly increased odds of receiving alcohol use treatment (aOR 2.90, 95% CI 1.22, 6.13; aOR 8.93, 95% CI 4.79, 16.40).

The average time between completion of the AUDIT‐C screening and the surgical procedure was similar across all alcohol risk categories. Individuals in the low‐risk group had a mean delay of 16.91 months (SD = 11.72), moderate‐risk group had 16.87 months (SD = 11.60), high‐risk group had 16.25 months (SD = 11.72), and severe‐risk group had 16.84 months (SD = 11.68). There was no statistically significant variation in timing from screening to procedure across AUDIT‐C risk levels (*p* = 0.30).

## DISCUSSION

In a national sample of participants in the *All of Us* Research Program undergoing surgical procedures that were screened for alcohol use with the AUDIT‐C, we assessed alcohol treatment rates 90 days peri‐procedurally. Our key findings indicate that psychotherapy or pharmacotherapy to reduce alcohol use is rarely initiated periprocedurally. Although 23.5%, 5.9%, and 4.1% of surgical patients qualified as moderate, high, and severe risk for unhealthy alcohol use, only 0.4%, 0.7%, and 4% of them received any alcohol treatment, respectively. Despite these overall low treatment rates, participants with higher risk for unhealthy alcohol use were more likely to receive treatment, thereby documenting the potential value of screening for unhealthy alcohol use in the perioperative environment. Any alcohol‐related treatment was also more likely among participants identified as male, diagnosed with anxiety or depression, and had a higher comorbidity burden, concordant with prior identified predictors of treatment utilization (Cohen et al., 2007).

Unhealthy alcohol use is one of the most common modifiable preoperative surgical risks (Khuri et al., 2008). However, clinicians place low priority on alcohol screening and interventions in the perioperative context (Fernandez et al., 2021). Our data show that neither pharmacological therapy nor psychotherapy is frequently offered during hospitalization episodes, upon discharge, or within 90 days of surgery. Similarly, in a sample of Medicare beneficiaries hospitalized for alcohol use disorder, only 0.7% were initiated on medical therapy within 2 days of discharge, and 1.3% were initiated within 30 days (Bernstein et al., 2023). The low sensitivity inherent to data registries is a well‐known limitation of the reliance upon electronic health record‐derived diagnosis and treatment codes that have historically been used for billing and reimbursement in the United States (Gianfrancesco & Goldstein, 2021). Furthermore, perioperative risks and procedure‐related diagnoses must account for temporal fluctuations, unlike more stable chronic comorbidities, which typically comprise comorbidity risk indices. However, previously reported low treatment rates are consistent with our findings.

In a systematic review of behavioral interventions, brief interventions aimed at reducing alcohol use preprocedurally were feasible and acceptable to patients, were linked to significant reductions in alcohol use in two of the studies, and reduced postoperative complications (Fernandez et al., 2015). Ongoing work to define feasible and acceptable interventions has found that both 10‐min brief advice and two 45‐min health coaching sessions preliminarily decreased average weekly alcohol use by 50% to 60% (Fernandez et al., 2022). Evidence‐based alcohol use‐specific interventions can decrease surgical complication rates if implemented in a timely and appropriate manner (Egholm et al., 2018). However, only one in six patients even discusses unhealthy alcohol use with a clinician, which leads to further underutilization of evidence‐based treatment (Fernandez et al., 2021; Michigan Overdose Prevention Engagement Network, 2025). According to the 2021 National Survey on Drug Use and Health, only 8.8% of people diagnosed with alcohol use disorder received any treatment (Substance Abuse and Mental Health Services Administration, 2022).

As evidenced, participants screened at high or severe risk for unhealthy alcohol use in this analysis were more likely to receive any therapy than those at low risk, thereby indicating the potential value of implementing the widespread use of validated screening tools. Indeed, medication initiation upon discharge has been shown to be feasible. A recent randomized clinical trial in 248 hospitalized patients diagnosed with AUD or with recent heavy drinking (≥5 drinks for males, ≥4 drinks for females) showed that patients who received either oral or injectable naltrexone on discharge experienced reduced heavy drinking days at 3‐month follow‐up (oral naltrexone: −38.4% absolute change, 95% CI, −125.0 to 48.2; extended‐release injectable naltrexone: −46.4% absolute change; 95% CI, −123.4 to 30.6) (Magane et al., 2025). Integrating alcohol screening and brief interventions or referral to therapy into routine perioperative practice represents an underutilized, yet promising opportunity to improve public health. In 2019, surgical patients accounted for 9 million of the hospital discharges in the United States (Weiss & Jiang, 2022). Further research to identify and remove barriers faced by patients and their clinicians to engage in alcohol‐specific perioperative care is needed.

### Limitations

There are limitations to this study. Inherent to the retrospective design is the potential for unaccounted confounding factors. We attempted to correct for unobserved bias using statistical techniques that adjusted for identifiable comorbidities (VanderWeele & Ding, 2017). Further, missing data rates can differ based on important socioeconomic variables, with historically underrepresented groups associated with higher missingness within electronic health records or research databases, especially in the *All of Us* Research Program (Cronin et al., 2023). We attempted to mitigate bias by not requiring case completion for inclusion in the analysis. Several socioeconomic variables are measured using a geographic area‐level approach (i.e., deprivation index), while outcomes are based on data from individuals. We note that area‐level data cannot account for the individual differences of the composite population, though local variability is captured more accurately by the granularity of the census tract‐level variables as opposed to larger geographic area measures (Trinidad et al., 2022). In addition, ICD codes for alcohol use disorder and related mental health comorbidities are unable to discern severity. Historically, ICD and Diagnostic and Statistical Manual of Mental Disorders criteria exhibited concordance; however, newer iterations diverge in their agreement on intermediate levels of clinical severity, which may affect data capture and representativeness (Lago et al., 2016). AUDIT‐C categories derived from patient‐reported surveys enhanced the granularity of the primary sample of concern: patients with patterns of alcohol use. We derived the AUDIT‐C scores from PPI codes, which captures data from the *All of Us* lifestyle surveys, rather than LOINC codes, which may result in an underrepresentation of clinician‐initiated screenings. Moreover, naltrexone is a nonselective opioid receptor antagonist that is prescribed to treat opioid use disorder, alcohol use disorder, and chronic pain (Goel et al., 2024; FDA, 2013). Here, we could not distinguish the indication for prescription, leading to a potential overestimation of the true treatment rate. Similarly, we were unable to distinguish indications for all psychotherapy codes. Regardless, 90‐day perioperative treatment rates for alcohol use remained rare.

## CONCLUSION

Patients undergoing a procedure and identified as severe risk for unhealthy alcohol consumption are more likely to receive alcohol treatment within 90 days of their procedure. Overall, and more importantly, alcohol treatment around the time of surgery is rare. Future work should focus on identifying and overcoming barriers to the implementation of alcohol use‐specific perioperative care.

## AUTHOR CONTRIBUTIONS

MLR: conceptualization, investigation, methodology, writing—original draft, review, and editing. MM: methodology, software, writing—original draft, visualization. SVM: formal analysis, data curation, writing—original draft. AJA: methodology, writing—review & editing. ACF: funding acquisition, investigation, writing—review & editing. APE: investigation, writing—review & editing. KB: conceptualization, methodology, investigation, supervision, project administration, funding acquisition, writing—review & editing.

## FUNDING INFORMATION

This work was supported in part by the National Institutes of Health (NIH), Award Number R34AA031020 (ACF, KB) and the Agency for Healthcare Research and Quality (AHRQ) Award Number R01HS027795 (KB). MR has received a research grant from the Society of Cardiovascular Anesthesiologists (SCA) In‐Training Grant unrelated to the present work. The content of this report is solely the responsibility of the authors and does not necessarily represent the official views of the NIH, AHRQ, and/or SCA. The NIH, AHRQ, and SCA were not involved in study design, collection, analysis, data interpretation, report writing, or the decision to submit the article for publication.

## CONFLICT OF INTEREST STATEMENT

The authors declare no competing interests.

## CLINICAL TRIAL NUMBER

Not applicable; the statistical plan was preregistered at Open Science Framework before data analysis (https://doi.org/10.17605/OSF.IO/WDY5K).

## Supporting information


Appendix S1.

## Data Availability

The data underlying this article were sourced from the *All of Us* Research Program's Controlled Tier Curated Data Repository (CDR v8). The *All of Us* Research Program at the National Institutes of Health (NIH) has a core value that states that data should be broadly accessible for research purposes. The program's dataset is stored on the Researcher Workbench, a secure cloud‐based platform, and is accessible to registered researchers. The *All of Us* Research Program Resource Access Board approved an exception to the Data and Statistics Dissemination Policy for this work.
