# Supplementary material for: Alcohol use‐specific treatment initiation among patients undergoing surgical procedures: A retrospective cohort analysis
Source: Alcohol Clin Exp Res (Hoboken). 2026 Jan 12;50(1):e70231. doi: 10.1111/acer.70231 (PMC12796779; doi:10.1111/acer.70231)
Supplement: Supplementary file 1 — Appendix S1. [file ACER-50-0-s001.docx]

Supplementary Material for the manuscript,

**Alcohol-Specific Treatment Initiation Among Patients Undergoing Surgical Procedures – A Retrospective Cohort Analysis Using the *All of Us* Database**

Table of Contents

[Post-hoc Power Analysis. 2](#_Toc209283993)

[Supplemental Figure A.1. Correlation plot. 3](#_Toc209283994)

[Supplemental Figure A.2. Unadjusted odds of receiving any alcohol treatment 90 days post-procedurally. 4](#_Toc209283996)

[Supplemental Table A.1: OMOP codes for identifying the AUDIT-C survey 5](#_Toc209283997)

[Supplemental Table A.2: OMOP codes for identifying AUD diagnosis 7](#_Toc209283998)

[Supplemental Table A.3. All of Us Controlled Tier Variable Definitions 8](#_Toc209283999)

*OMOP procedural and treatment code identifiers are available upon request.

# Post-hoc Power Analysis.

A simulation-based power analysis was conducted to evaluate the power of our multivariable logistic regression model. The simulation explicitly incorporated the observed covariance structure among all predictors—including the significant correlations between area-level socioeconomic variables—by using the estimated correlation matrix from our full dataset (N=7,142) to generate synthetic data. Monte Carlo simulations (n=1000 iterations) were performed, which involved generating datasets, fitting the full model, and testing the significance (α=0.05) of the AUDIT-C score. The analysis indicated that the current sample size provided 99.5% power to detect the effect of the primary predictor. The effect is robust and could be reliably detected with a smaller sample.

# Supplemental Figure A.1. Correlation plot.


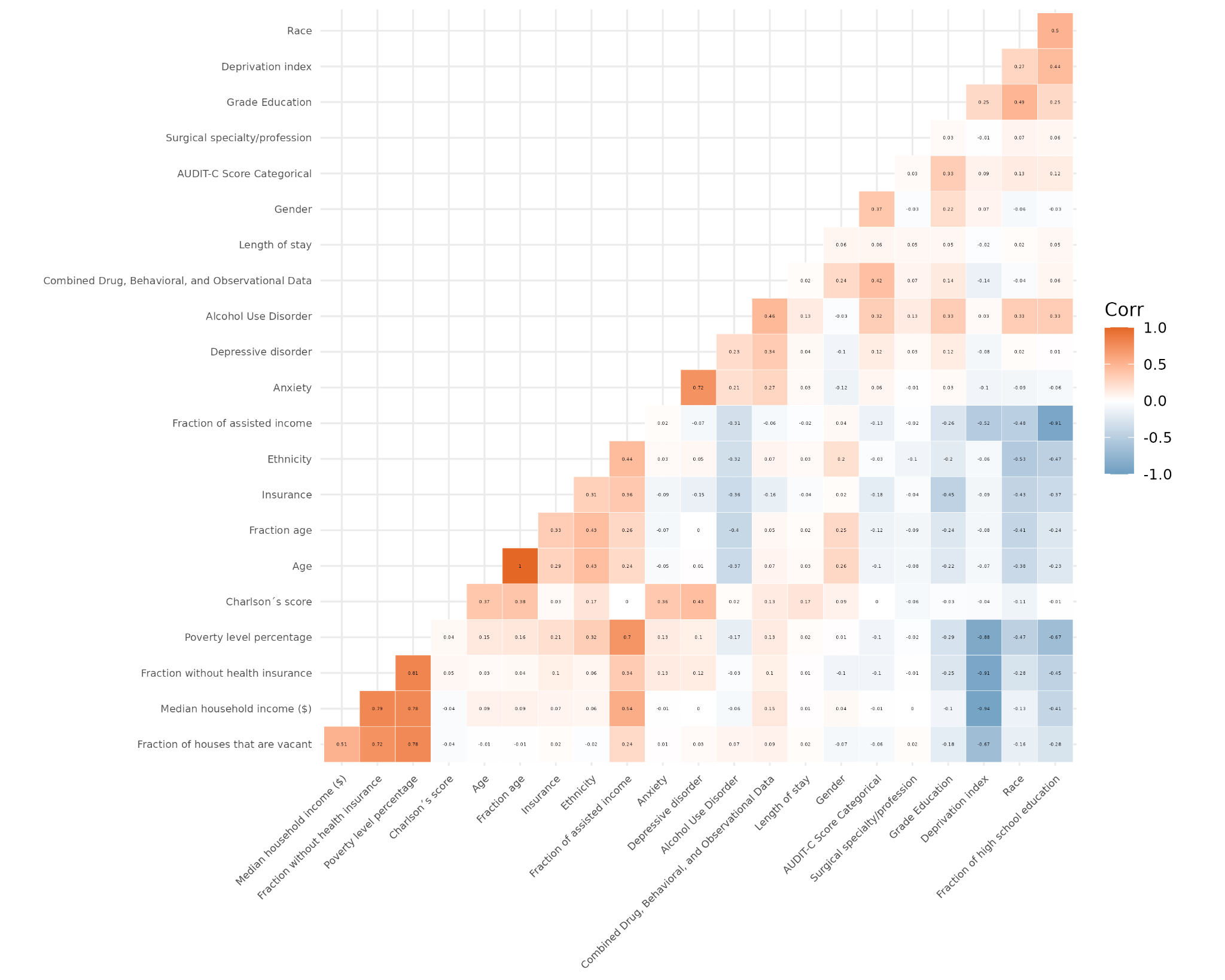


# *Variable definitions and provenance are reported in Supplementary Table 6. Combined drug, behavioral, and observational data refers to the “any alcohol-specific therapy” category.

# Supplemental Figure A.2. Unadjusted odds of receiving any alcohol treatment 90 days post-procedurally.

##

# Supplemental Table A.1: OMOP codes for identifying the AUDIT-C survey

| **OMOP code** | **Question** | **OMOP code** | **Answer** |
| --- | --- | --- | --- |
| 1586207 | Alcohol: Average Daily Drink Count | Alcohol: Average Daily Drink Count | PMI: Skip |
| 1586207 | Alcohol: Average Daily Drink Count | Alcohol: Average Daily Drink Count | PMI: Prefer Not To Answer |
| 1586207 | Alcohol: Average Daily Drink Count | Alcohol: Average Daily Drink Count | Average Daily Drink Count: 1 or 2 |
| 1586207 | Alcohol: Average Daily Drink Count | Alcohol: Average Daily Drink Count | Average Daily Drink Count: 3 or 4 |
| 1586207 | Alcohol: Average Daily Drink Count | Alcohol: Average Daily Drink Count | Average Daily Drink Count: 5 or 6 |
| 1586207 | Alcohol: Average Daily Drink Count | Alcohol: Average Daily Drink Count | Average Daily Drink Count: 10 or More |
| 1586201 | Alcohol: Drink Frequency Past Year | Alcohol: Drink Frequency Past Year | PMI: Skip |
| 1586201 | Alcohol: Drink Frequency Past Year | Alcohol: Drink Frequency Past Year | PMI: Prefer Not To Answer |
| 1586201 | Alcohol: Drink Frequency Past Year | Alcohol: Drink Frequency Past Year | Drink Frequency Past Year: Never |
| 1586201 | Alcohol: Drink Frequency Past Year | Alcohol: Drink Frequency Past Year | Drink Frequency Past Year: 2 to 3 Per Week |
| 1586201 | Alcohol: Drink Frequency Past Year | Alcohol: Drink Frequency Past Year | Drink Frequency Past Year: Monthly Or Less |
| 1586201 | Alcohol: Drink Frequency Past Year | Alcohol: Drink Frequency Past Year | Drink Frequency Past Year: 2 to 4 Per Month |
| 1586201 | Alcohol: Drink Frequency Past Year | Alcohol: Drink Frequency Past Year | Drink Frequency Past Year: 4 or More Per Week |
| 1586213 | Alcohol: 6 or More Drinks Occurrence | Alcohol: 6 or More Drinks Occurrence | PMI: Skip |
| 1586213 | Alcohol: 6 or More Drinks Occurrence | Alcohol: 6 or More Drinks Occurrence | PMI: Prefer Not To Answer |
| 1586213 | Alcohol: 6 or More Drinks Occurrence | Alcohol: 6 or More Drinks Occurrence | 6 or More Drinks Occurrence: Daily |
| 1586213 | Alcohol: 6 or More Drinks Occurrence | Alcohol: 6 or More Drinks Occurrence | 6 or More Drinks Occurrence: Weekly |
| 1586213 | Alcohol: 6 or More Drinks Occurrence | Alcohol: 6 or More Drinks Occurrence | 6 or More Drinks Occurrence: Monthly |
| 1586213 | Alcohol: 6 or More Drinks Occurrence | Alcohol: 6 or More Drinks Occurrence | 6 or More Drinks Occurrence: Less Than Monthly |
| 1586213 | Alcohol: 6 or More Drinks Occurrence | Alcohol: 6 or More Drinks Occurrence | 6 or More Drinks Occurrence: Never In Last Year |
| 1586207 | Alcohol: Average Daily Drink Count | Alcohol: Average Daily Drink Count | Average Daily Drink Count: 7 to 9 |

# Supplemental Table A.2: OMOP codes for identifying AUD diagnosis

| **OMOP code** | **AUD diagnosis** |
| --- | --- |
| 45757131 | Alcohol dependence in childbirth |
| 4218106 | Alcoholism |
| 4338024 | Absinthe addiction |
| 45757093 | Alcohol dependence in pregnancy |
| 37018356 | Moderate alcohol dependence |
| 4152165 | Nondependent alcohol abuse |
| 37017563 | Severe alcohol dependence |
| 43695 | Continuous chronic alcoholism |
| 4109691 | Persistent alcohol abuse |
| 44128 | Nondependent alcohol abuse in remission |
| 43524 | Alcohol dependence |
| 43553 | Nondependent alcohol abuse, continuous |
| 43901 | Chronic alcoholism in remission |
| 43553 | Episodic chronic alcoholism |
| 37017329 | Mild alcohol dependence |
| 44069 | Nondependent alcohol abuse, episodic |
| 43375 | Alcohol abuse |

#

# Supplemental Table A.3. All of Us Controlled Tier Variable Definitions

| Field Name | Data Provenance | Description |
| --- | --- | --- |
| Education | The Basics Survey Module (LOINC) | Highest grade or level of schooling completed (SAMHSA) |
| Insurance | The Basics Survey Module (LOINC) | Categorical insurance type |
| Fraction of poverty | American Community Survey | Fraction of the population with income in the past 12 months below the poverty level |
| Median household income | American Community Survey | Median household income in the past 12 months in 2015 inflation-adjusted dollars |
| Assisted income | American Community Survey | Fraction of households receiving public assistance income or food stamps or SNAP in the past 12 months |
| Uninsured population | American Community Survey | Fraction of population with no health insurance coverage |
| Vacant housing | American Community Survey | Fraction of houses that are vacant |
| Fraction of high school education | American Community Survey | Fraction of adults aged 25 and older with at least a high school education (including GED equivalency) |
| Deprivation index | American Community Survey | A deprivation index for each census tract in the United States based on a principal components analysis of six different 2015 ACS measures; rescaling and normalizing forces the index to range from 0 to 1, with a higher index being more deprived |
| Length of stay | OMOP CDM Metadata (Externally generated): Visit occurrence table | Represents the number of days in a patient stay, calculated as the difference between discharge and admission dates. |
| Alcohol Use Disorder (AUD) | EHR: Condition occurrence table | ICD-10 diagnosis code. Diagnosis, signs, or symptoms of AUD observed by providers or reported by patients. |
| Pharmacotherapy | OMOP CDM Metadata (Externally generated): Drug exposure table | Drug exposure is determined by clinical events related to orders, prescriptions written, pharmacy dispensing, procedural administrations, and other patient-reported data. |
| Behavioral therapy | OMOP CDM Metadata (Externally generated): procedure occurrence table | Procedures performed by a healthcare provider related to referrals to psychotherapy, brief interventions, or detoxification programs for therapeutic purposes. |
| Observational Variable | OMOP CDM Metadata (Externally generated): observation table | Clinical facts about referrals to psychotherapy, brief interventions, or detoxification programs gathered during examination, questioning, or survey methodology. |
| Any treatment | OMOP CDM Metadata (Externally generated) | A combination of pharmacotherapy, behavioral strategies, and observational variables, representing any alcohol-specific treatment. |
| AUDIT-C: Alcohol Use Disorders Identification Test-Consumption | PPI: survey data table | The survey data includes a complete set of items from the AUDIT-C instrument. |

STROBE Statement—checklist of items that should be included in reports of observational studies

|  | Item No | Recommendation | Page  No |
| --- | --- | --- | --- |
| **Title and abstract** | 1 | (*a*) Indicate the study’s design with a commonly used term in the title or the abstract | 1 |
|  |  | (*b*) Provide in the abstract an informative and balanced summary of what was done and what was found | 3 |
| Introduction | | | |
| Background/rationale | 2 | Explain the scientific background and rationale for the investigation being reported | 4 |
| Objectives | 3 | State specific objectives, including any prespecified hypotheses | 5 |
| Methods | | | |
| Study design | 4 | Present key elements of study design early in the paper | 6 |
| Setting | 5 | Describe the setting, locations, and relevant dates, including periods of recruitment, exposure, follow-up, and data collection | 6 |
| Participants | 6 | (*a*) *Cohort study*—Give the eligibility criteria, and the sources and methods of selection of participants. Describe methods of follow-up  *Case-control study*—Give the eligibility criteria, and the sources and methods of case ascertainment and control selection. Give the rationale for the choice of cases and controls  *Cross-sectional study*—Give the eligibility criteria, and the sources and methods of selection of participants | 6 |
|  |  | (*b*) *Cohort study*—For matched studies, give matching criteria and number of exposed and unexposed  *Case-control study*—For matched studies, give matching criteria and the number of controls per case | N/A |
| Variables | 7 | Clearly define all outcomes, exposures, predictors, potential confounders, and effect modifiers. Give diagnostic criteria, if applicable | 7 |
| Data sources/ measurement | 8* | For each variable of interest, give sources of data and details of methods of assessment (measurement). Describe comparability of assessment methods if there is more than one group | 8 |
| Bias | 9 | Describe any efforts to address potential sources of bias | 9-10 |
| Study size | 10 | Explain how the study size was arrived at | 9 |
| Quantitative variables | 11 | Explain how quantitative variables were handled in the analyses. If applicable, describe which groupings were chosen and why | 8/9 |
| Statistical methods | 12 | (*a*) Describe all statistical methods, including those used to control for confounding | 8/9 |
|  |  | (*b*) Describe any methods used to examine subgroups and interactions | 10 |
|  |  | (*c*) Explain how missing data were addressed | 9 |
|  |  | (*d*) *Cohort study*—If applicable, explain how loss to follow-up was addressed  *Case-control study*—If applicable, explain how matching of cases and controls was addressed  *Cross-sectional study*—If applicable, describe analytical methods taking account of sampling strategy | N/A |
|  |  | (*e*) Describe any sensitivity analyses | 10 |

| Results | | | |
| --- | --- | --- | --- |
| Participants | 13* | (a) Report numbers of individuals at each stage of study—eg numbers potentially eligible, examined for eligibility, confirmed eligible, included in the study, completing follow-up, and analysed | 11/Fig1 |
|  |  | (b) Give reasons for non-participation at each stage |  |
|  |  | (c) Consider use of a flow diagram | Fig 1 |
| Descriptive data | 14* | (a) Give characteristics of study participants (eg demographic, clinical, social) and information on exposures and potential confounders | Table 1 |
|  |  | (b) Indicate number of participants with missing data for each variable of interest | 9 |
|  |  | (c) *Cohort study*—Summarise follow-up time (eg, average and total amount) |  |
| Outcome data | 15* | *Cohort study*—Report numbers of outcome events or summary measures over time | 11-12 |
|  |  | *Case-control study—*Report numbers in each exposure category, or summary measures of exposure |  |
|  |  | *Cross-sectional study—*Report numbers of outcome events or summary measures |  |
| Main results | 16 | (*a*) Give unadjusted estimates and, if applicable, confounder-adjusted estimates and their precision (eg, 95% confidence interval). Make clear which confounders were adjusted for and why they were included | 11-12 |
|  |  | (*b*) Report category boundaries when continuous variables were categorized | 11-12 |
|  |  | (*c*) If relevant, consider translating estimates of relative risk into absolute risk for a meaningful time period |  |
| Other analyses | 17 | Report other analyses done—eg analyses of subgroups and interactions, and sensitivity analyses | 12 |
| Discussion | | | |
| Key results | 18 | Summarise key results with reference to study objectives | 13-14 |
| Limitations | 19 | Discuss limitations of the study, taking into account sources of potential bias or imprecision. Discuss both direction and magnitude of any potential bias | 14-15 |
| Interpretation | 20 | Give a cautious overall interpretation of results considering objectives, limitations, multiplicity of analyses, results from similar studies, and other relevant evidence | 15 |
| Generalisability | 21 | Discuss the generalisability (external validity) of the study results | 14-15 |
| Other information | | | |
| Funding | 22 | Give the source of funding and the role of the funders for the present study and, if applicable, for the original study on which the present article is based | 2 |
